# Molecular, Physicochemical and Rheological Characteristics of Introgressive *Triticale/Triticum monococcum* ssp. *monococcum* Lines with Wheat 1D/1A Chromosome Substitution

**DOI:** 10.3390/ijms140815595

**Published:** 2013-07-26

**Authors:** Bolesław P. Salmanowicz, Monika Langner, Halina Wiśniewska, Barbara Apolinarska, Michał Kwiatek, Lidia Błaszczyk

**Affiliations:** Institute of Plant Genetics, Polish Academy of Sciences, Strzeszyńska Str. 34, Poznań 60-479, Poland; E-Mails: bsal@igr.poznan.pl (B.P.S.); mlan@igr.poznan.pl (M.L.); bapo@igr.poznan.pl (B.A.); mkwi@igr.poznan.pl (M.K.); lgol@igr.poznan.pl (L.B.)

**Keywords:** bread-making quality, chromosome substitution, glutenin, *Triticum monococcum*, *Triticale*

## Abstract

Three sets of hexaploid introgressive triticale lines, with *Triticum monococcum* ssp. *monococcum* (cultivated einkorn wheat) genes and a bread wheat chromosome 1D substituted for chromosome 1A, and one set of secondary triticale lines were evaluated for grain and flour physicochemical and dough rheological characteristics in two generations (F7 and F8). Genomic *in situ* hybridization (GISH) and fluorescence *in situ* hybridization (FISH) confirmed the 1D/1A chromosome substitution. The presence or absence of einkorn high-molecular-weight (HMW) glutenin subunits and the wheat *Glu-D1d* locus encoding the 5 + 10 subunits was assessed by sodium dodecyl sulfate-polyacrylamide gel electrophoresis (SDS-PAGE), capillary zone electrophoresis, and allele-specific molecular markers. Significant differences were found among physicochemical properties (with the exception of the Hagberg falling number) of all introgressive *Triticale/T. monococcum* lines and the secondary triticale lines. The wheat 1D/1A chromosome substitution also affected these properties. The results showed that in all introgressive triticale lines, the protein and gluten content, Zeleny sedimentation value, and water absorption capacity, were increased. The rheological parameters estimated using micro-farinograph, reomixer, and Kieffer dough extensibility systems also showed an appreciable increase in dough-mixing properties, maximum resistance to extension (R_max_), and dough extensibility. Introgressive *Triticale/T. monococcum* lines with 5 + 10 subunits have particularly favorable rheological parameters. The results obtained in this study suggest that the cultivated einkorn genome A^m^, in the context of hexaploid secondary triticale lines and with a wheat 1D/1A substitution, has the potential to improve gluten polymer interactions and be a valuable genetic resource for triticale quality improvement.

## 1. Introduction

Triticale (x *Triticosecale* Wittmack, *Triticale*), created by crossing wheat (*Triticum aestivum* L.) and rye (*Secale cereale* L.), combines many desirable traits of both parents. This crop can produce a higher grain yield and total biomass production than any other cereal over a wide range of soil and climatic conditions. The biological value of triticale makes it suitable for use as animal feed and for human nutrition [1]. Present-day cultivated triticale is hexaploid (AABBRR) and differs from bread wheat (AABBDD) by the presence of the R genome of rye, which replaces the wheat D genome. This replacement not only reduces the total gluten content in triticale grains, but also removes genetic loci encoding important glutenins (*Glu-D1* and *Glu-D3*) and introduces rye secalin loci, which leads to a decrease in the end-use quality of triticale flour [2,3]. Despite partial homology, rye secalins, which are encoded by the *Sec-1*, *Sec-2*, and *Sec-3* loci, differ significantly from wheat storage proteins (glutenins and gliadins) with respect to structural and quantitative parameters that are important for the formation and properties of wheat gluten [3,4]. In *T. aestivum*, the *Glu-D1* locus on chromosome 1D, which encodes the high molecular weight (HMW) glutenin subunits 5 + 10, is considered to be a particularly important contributor to bread-making quality. As the initial wheat parent of hexaploid triticale originates from tetraploid wheat, and does not contain the D genome, triticale flour generally lacks the quality required for bread-making.

A number of differences in dough rheological parameters, and during the baking process, appear in common triticale varieties containing a complete R genome. To improve the bread-making quality of hexaploid triticale, attempts to substitute or add chromosome 1D have been made. These studies showed that the presence of chromosome 1D had a significant and positive effect on the sedimentation value of hexaploid lines [5,6]. However, no significant differences were found in the bread-making quality of triticale lines carrying either *a* or *d Glu-D1* alleles (with HMW-GS 2 + 12 and 5 + 10, respectively), which are associated with contrasting (low and high) baking qualities in wheat. The 1D/1A substitution appears to have minimal effect on agronomic performance, while 1D/1B and 1D/1R substitutions showed considerable yield loss [6–8]. To improve the bread-making quality of hexaploid triticale, a series of cytogenetically engineered 1R chromosomes, with various wheat-like genetic constructs at the gluten loci, was performed [4,8–12]. Four types of chromosomes (Valdy, FC1, FC2, and RM) with multi-breakpoint translocations were studied. Triticale lines with FC2 chromosomes, which differ from bread wheat only by the absence of the *Gli-D2* locus and the presence of the *Sec-2* locus, appeared to be the most suitable for triticale breeding [12].

Cultivated einkorn (*Triticum monococcum* L. ssp*. monococcum*, 2*n* = 2*x* = 14, A^m^A^m^) is closely related to *Triticum urartu* (2*n* = 2*x* = 14, A^u^A^u^), which is one of the progenitors of hexaploid bread wheat. Various einkorn genetic loci were successfully introgressed into hexaploid triticale to provide resistance to leaf rust and powdery mildew [13,14] and to prevent pre-harvest sprouting [15]. In contrast, cultivated einkorn shows a considerable number of allelic variants of glutenins that are not reported in hexaploid wheat and triticale, and it is therefore a potentially important source of novel genes for improving wheat and triticale quality [16–20]. It has been shown that the bread-making quality of wheat is improved by introgression of the genes encoding the high molecular weight (HMW) glutenin subunits from *T. monococcum* [21,22]. Additionally, y-type subunits encoded by the *Glu-A1y* locus are expressed in a considerable number of einkorn accessions [23–26], but these subunits are absent in hexaploid triticale. Furthermore, in hexaploid triticale the *Glu-A1x* locus is characterized by a small number of alleles (1, 2* and Null) [27]. The transfer of new subunits from einkorn to triticale varieties may improve the protein content and bread-making processing quality. To our knowledge, there are no reports of a molecular and rheological analysis of introgressive triticale lines with the cultivated einkorn genome A^m^ and a bread wheat 1D/1A chromosome substitution.

The aim of the study was to investigate the influence of a 1D/1A chromosome substitution in hexaploid introgressive *Triticale/T. monococcum* lines on grain quality and dough rheological properties using the criteria for bread-making quality in wheat. Simultaneously, we describe the potential of the *T. monococcum* genome to improve the end-product quality of secondary triticale.

## 2. Results and Discussion

### 2.1. Cytogenetic and Molecular Analyses

The screening genomic *in situ* hybridization (GISH) experiment revealed that 6 of 36 lines carried substitutions of chromosomes of “D” subgenome. These lines were chosen to fluorescence *in situ* hybridization (FISH) experiment in order to identify chromosomes.

**Figure 1:**
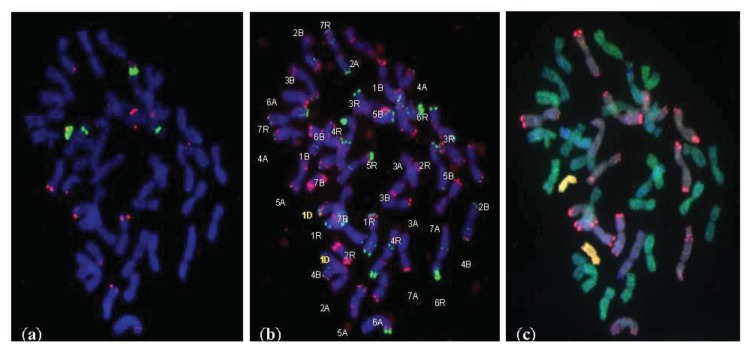
Substitution (1D/1A) line, 2*n* = 42 chromosomes. (**a**) FISH with 5S rDNA (red) and 35S rDNA (green); (**b**) FISH with *pAs1* (red) and *pSc 119.2* (green). The chromosomes were counterstained with 4′,6-diamidino-2-phenylindole (DAPI, blue); (**c**) mc GISH with total genomic DNA from rye—R genome (red), total genomic DNA from *Triticum monococcum*—A genome (green) and total genomic DNA from *Aegilops tauschii*—D genome (yellow) with blocking genomic DNA of *Aegilops speltoides*—B genome (DAPI, blue).

FISH experiments with 5S rDNA, 35S rDNA, pSc119.2, and pAs1 probes provided specific patterns of signals that allowed to identification of six lines with chromosome 1D substitution (Figure 1a–c). The presence of D chromatin in triticale genome can increase the utility of this cereal.

**Figure 2:**
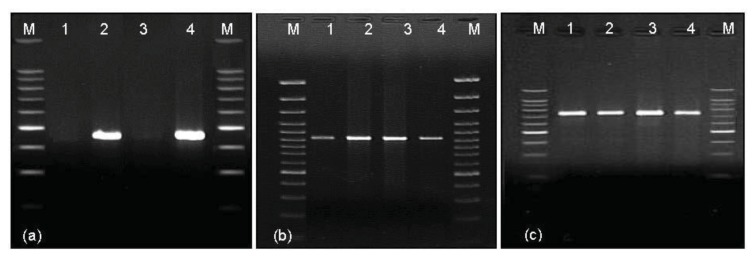
Dominant PCR markers for triticale HMW glutenin subunits (**a**) Dx5/Dx2; (**b**) AxNull; and (**c**) By20*. Primer set GS I produces a 450 bp fragment in triticale lines that have the *Glu-D1d* (Dx5) allele. The PCR product for the Glu-*A1c* (AxNull) allele is 920 bp and that for the By20* subunit is 750 bp. Lanes, M, 100-bp DNA Ladder Plus (Promega); 1, Glu-Sec group; 2, Glu-Sec + Tm; 3, Glu-Sec + 1D; 4, Glu-Sec + Tm + 1D group.

Molecular analyses confirmed the occurrence of the 1D/1A substitution as part of the introgressive triticale lines. PCR with the primer set GS I gave a band of 450 bp (Figure 2a, lanes 2, 4), which is an allele-specific marker for the *Glu-D1d* allele encoding the Dx5 subunit [28], in six lines, confirming the cytogenetic analysis.

Additionally, an amplified product of 920 bp, which is characteristic of the wheat *Glu-A1c* allele (AxNull) [29], was observed for all triticale samples when the GS II primer set was used (Table 1). The presence of the GS III primer pair, which is an allele-specific marker for the triticale subunit By20* [30,31], in the reaction mixture, gave rise to one band of 750 bp (Figure 2c) in all triticale samples. The allelic composition encoding subunit pair 6.8 + 20* frequently appears among European triticale cultivars [27,32].

### 2.2. Variation of HMW Glutenin Subunit Composition of Introgressive *Triticale* Lines

**Figure 3:**
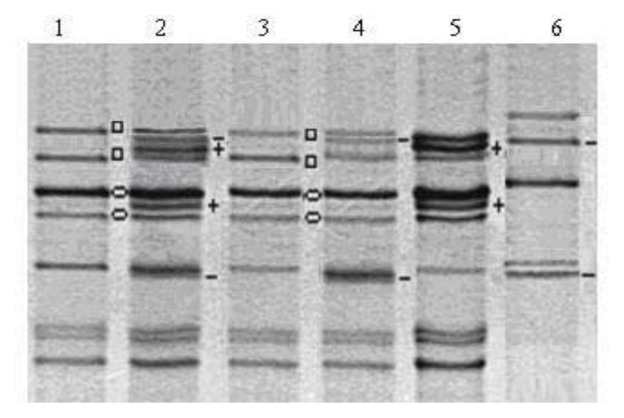
SDS-PAGE patterns of wheat and einkorn HMW glutenin and rye secalins in introgressive *Triticale/Triticum monococcum* lines, with four characteristic subunit sets; ○ Glu (wheat subunits 6.8 + 20*), □ Sec (rye subunits 2r + 5.3r), + Tm (einkorn subunits 5.2 m + 14 m), − 1D (wheat subunits 5 + 10). Lanes: 1, standard triticale cv. Gabo (Null/6.8 + 20*, 2r + 5.3r); 2, Glu-Sec + Tm + 1D; 3, Glu-Sec; 4, Glu-Sec + 1D; 5, Glu-Sec + Tm; 6, standard bread wheat cv. Panda (1/7 + 9/5 + 10).

HMW glutenin and secalin compositions have been widely used to assess the genetic diversity and grain quality of wheat and triticale. The HMW-GS of wheat are major determinants of bread-making quality; in particular, the subunits 5 + 10 and subunit 1 and 2* have high quality scores [33]. sodium dodecyl sulfate-polyacrylamide gel electrophoresis (SDS-PAGE) migration patterns of HMW glutenin and secalin subunits (HMW-GS and HMW-SS) from representative samples are shown in Figure 3, together with those from standard bread wheat (cv. Panda) and triticale (cv. Gabo) cultivars. All analyzed lines carried wheat x-type subunit 6.8 and y-type subunit 20*, encoded by the *Glu-B1* locus, and rye subunits 2r and 5.3r, encoded by the *Sec-3* (*Glu-R1*) locus, but did not have the wheat glutenin subunit encoded by the *Glu-A1* locus [*Glu-1c* (Null) allele] (Figure 3). This HMW glutenin and secalin allelic composition is common among triticale cultivars [32]. As observed in the cytological and molecular studies shown above, six lines carried the wheat x-type subunit 5 and y-type subunit 10, which are encoded by genes located on chromosome 1D (Figure 3, lanes 2, 4). SDS-PAGE analysis also revealed bands for two einkorn HMW glutenin subunits in 19 lines, an x-type subunit, which migrates more slowly than the rye subunit 5.3r, and a y-type subunit, which migrates more quickly than the wheat subunit 14. These two extra subunits were encoded by genes located on chromosome 1A^m^ and were designated 5.2 m and 14 m, respectively (Figure 3, lanes 2, 5).

The analyzed lines can be divided into four groups on the basis of their HMW-GS and -SS composition, 14 lines are typical secondary hexaploid triticale (Glu-Sec), three lines have additional wheat subunits 5 and 10 (Glu-Sec + 1D), and 19 lines are secondary hexaploid triticale with two additional subunits (5.2 m + 14 m), although three of them also have subunits 5 and 10 (Glu-Sec + Tm and Glu-Sec + Tm + 1D, respectively) (Table 2).

**Figure 4:**
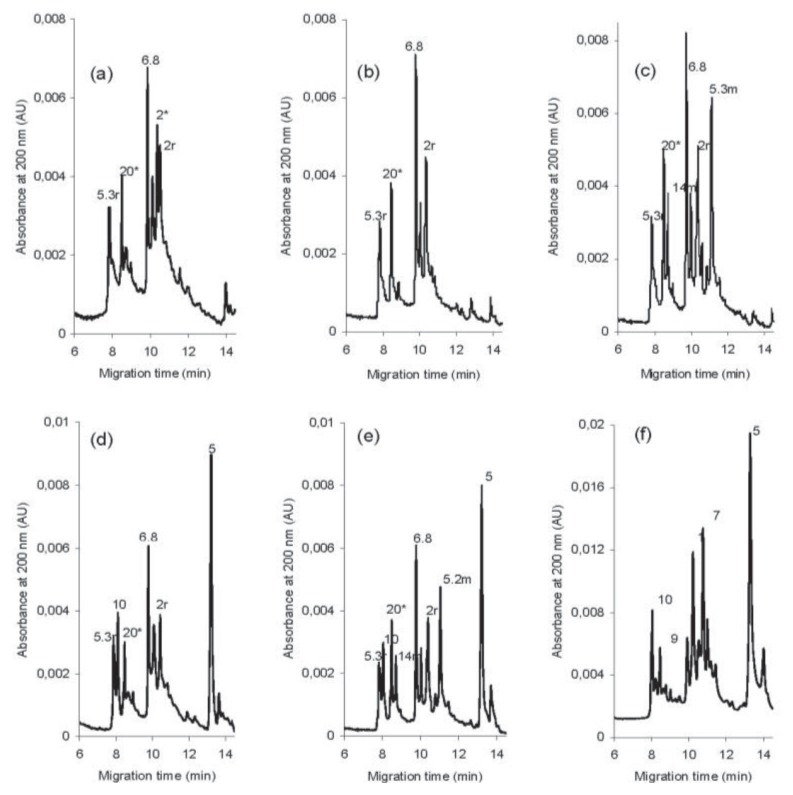
CZE electrophorograms of nonalkylated HMW proteins from four distinct introgressive *Triticale/Triticum monococcum* groups with various HMW-GS and HMW-SS compositions and reference triticale and wheat cultivars. (**a**) standard triticale (cv. Lasko; 2*/6.8 + 20*/2r + 5.3r); (**b**) Glu + Sec (N/6.8 + 20*/2r + 5.3r); (**c**) Glu + Sec + Tm (N/6.8 + 20*/2r + 5.3r; 5.2 m + 14 m); (**d**) Glu + Sec + 1D (N/6.8 + 20*/2r + 5.3r; 5 + 10); (**e**) Glu + Sec + Tm + 1D (N/6.8 + 20*/2r + 5.3r; 5.2 m + 14 m; 5 + 10); (**f**) Standard wheat (cv. Panda) (1/7 + 9/5 + 10). The HMW-SS and HMW-Tm peaks are additionally indicated by the letter “*r*” and “*m*”, respectively, at the end of sing. The proteins were separated at 10 kV and 40 °C with a 75 mM iminodiacetic acid (IDA), 0.15% poly(ethylene oxide) (PEO) with a molecular weight of 8,000,000, 0.05% 26 mM SB3-12, and 20% (*v*/*v*) acetonitrile.

The detailed identification of the HMW subunit fractions (HMW-GS and -SS) of all 36 samples was performed by capillary zone electrophoresis (CZE). The presence of both wheat and rye HMW proteins interferes with the identification of individual HMW subunits in triticale by the SDS-PAGE method (wheat and rye HMW proteins have similar molecular weights and electrophoretic mobilities, but appear at varying levels). As revealed in earlier investigations by Salmanowicz [32,34], triticale HMW-GS and -SS can be easily discriminated by CZE. As shown in Figure 4, from four to eight major protein peaks with migration times in the range of 7.8 to 13.2 min are observed in the CZE electrophoretic profiles of the four different triticale groups. The typical triticale Glu-Sec group shows four major protein peaks in its CZE profiles, two wheat HMW-GS (the major protein peaks of subunits 6.8 and 20*, with migration times (*t*_m_) of 9.81 and 8.48 min, respectively), and two rye HMW-SS (the major peaks of subunits 2r and 5.3r, with *t*_m_ of 10.36 and 7.82 min, respectively). The introgressive triticale Glu-Sec + Tm group shows six major protein peaks in its CZE profiles, two wheat subunits (6.8 + 20*), two rye HMW-SS (2r + 5.3r), and two einkorn HMW-GS (the major peaks of subunits 5.2 m and 14 m, with t_m_ of 11.04 and 8.71 min, respectively). However, the triticale Glu-Sec + 1D group also revealed two additional wheat peaks (the major peaks of subunits 5 and 10, with t_m_ of 13.19 and 8.02 min, respectively).

### 2.3. Effects of HMW Glutenin Subunit Composition on Physicochemical Properties of Introgressive Triticale Lines

The physicochemical characteristics of introgressive secondary triticale flours with various HMW-GS and -SS compositions at two generations (F7 and F8) are presented in Table 3. A comparison of the means showed that only two of the eight evaluated quality parameters (grain moisture content and falling number) were similar (*p* < 0.05) across the four distinct triticale groups. Generally, the physicochemical characteristics were higher for all the introgressive *Triticale/T. monococcum* lines than for the typical triticale (Glu-Sec).

The Glu + Sec group showed the highest thousand kernel weight (mean 41.5 g), whereas considerably lower values, in the range of 36.1–38.91 g (mean 37.6 g), were observed for introgressive lines with the *T. monococcum* genome (the Glu +Sec+ Tm and Glu + Sec + Tm + 1D groups). A decrease in the thousand-kernel weight in wheat and triticale is often observed in genotypes with chromosomal translocations or substitutions [7,8,11].

Protein and gluten content are considered important triticale quality characteristics that provide an indication of the final quality and functionality. The protein content of the analyzed material ranged from 10.3 to 11.9 g/100 g for the next two generations. Statistical analysis revealed that the protein and gluten contents of the introgressive lines were significantly higher than those of the typical triticale. These two quality traits can vary significantly for given cultivars, depending on the environmental conditions under which they are grown, but these traits are also under genetic control [35]. In this study, the protein and gluten content of all lines from the 2009/2010 vegetation season (generation F7) were higher than those of lines from the year 2010/2011 (F8). The ash content in all analyzed flours was not significantly different (*p* < 0.05) and amounted to approximately 0.85 g/100 g. The starch content in the analyzed material varied significantly, from 59.1 to 62.9 g/100 g. Flour obtained from lines with the 1D/1A chromosome substitution showed the lowest starch content, lower than that of flour from introgressive lines with only the *T. monococcum* genome. The highest ZSL values, which are related to protein structures that are important for dough formation, were obtained in introgressive lines from the Glu-Sec + Tm + 1D and Glu-Sec + 1D groups. The average value was 21.9 mL in the typical triticale, 26.7 mL in the introgressive lines with the Dx5 + Dy10 subunits, and 27.3 mL in the *Triticale/T. monococcum* lines with the 1D/1A chromosome substitution. These results are in agreement with those reported by Lafferty and Lelley [6], and Martinek *et al.* [11], for triticale lines with a HMW glutenin *Glu-D1d* allele translocation or a substitution of bread wheat chromosome 1D.

The Hagberg falling number (FN) is still one of most the commonly used methods for determining kernel damage caused by α-amylase activity [11,36–38]. Excessive levels of α-amylase are most commonly produced in pre-harvest sprouting or as late-maturity α-amylase. Generally, hexaploid secondary triticales are characterized by very low FN (60–100 s) in comparison with bread wheat (above 200 s). The FN data obtained in this study indicated that the introduction of the A^m^ genome and/or 1D/1A substitution into hexaploid triticale did not cause fundamental changes in enzyme activity. The mean FN values ranged from 64 to 71 s in the analyzed samples from two generations and were not statistically significantly different (*p* < 0.05). However, a slight increase in the falling number value was detected for flour extracted from the introgressive lines with the introduction of the *T. monococcum* genome. This increase in the FN in the introgressive triticale lines may be the result of the observed increase in grain protein content. Kindred and coworkers [37], and Ross *et al.* [38], have demonstrated that grain protein content and FN are positively correlated, and the PC can substantially modulate the FN. Two-fold higher FN values in some introgressive *Triticale/T. monococcum* lines showed earlier by Sodkiewicz [36], not fully observed in our experiments can be due to effect of gene-environment interaction. The lowered activity of hydrolytic enzymes reported for some new triticale cultivars (FN > 100 s) indicates that this trait, which is very important for bread-making quality, can be improved via triticale breeding [39].

### 2.4. Effects of HMW Glutenin Subunit Composition on Rheological Properties of *Triticale* Flour

The rheological properties of dough are critical to bread-making quality. Several micro-scale techniques (*ca.* 10 g flour) are employed to evaluate wheat and triticale lines for properties that may influence the final quality. Commonly used tools are the micro-farinograph, mixograph, and micro-scale extension tests, which measure the resistance of the dough to mixing after the addition of water. Key measurements include the amount of water needed to obtain the optimal resistance of the dough during mixing, the time required to achieve the optimal resistance, and the time that dough will remain at the peak resistance before breakdown. Strong dough with a high tolerance during mixing and good water absorption is generally associated with good bread-making quality, at least for wheat flour [40].

#### 2.4.1. Quality of Dough Mixing Properties

The farinograph test parameters of triticale flour from the four different groups were measured to evaluate changes in water absorption, dough development time, dough stability time, and degree of softening. In two triticale generations (F7 and F8), the water absorption values were in the range of 65.1% to 68.9% (Table 4).

The introgressive triticale lines showed significantly (*p* < 0.05) higher water absorption capacities than the typical triticale. The higher values of this parameter for the triticale lines with the A^m^ genome and/or the 1D/1A substitution (with wheat and einkorn HMW-GS) were an artifact of the significantly higher flour protein and gluten contents. The mean dough development time, dough stability, and degree of softening were significantly (*p* < 0.05) higher for the introgressive lines from both generations than for the typical triticale line. The triticale group with the 1D/1A substitution (Glu-Sec + 1D) has a decrease in these farinograph parameters, similar to the lines with the introduced A^m^ genome (Glu-Sec + Tm). For the introgressive *Triticale/T. monococcum* lines with the 1D/1A chromosome substitution, we detected almost two-fold increases in these parameters over those of the secondary triticale. Additionally, previous studies by Lafferty and Lelley [6], Lukaszewski [4], and Martinek *et al*. [11] showed a positive influence of wheat 1D substitution or translocation (Dx5 + Dy10) on the bread-making quality of triticales. In summary, the farinograph parameter values detected for all three introgressive triticale lines were characteristic of weak flour (for example, high degree of softening values (above 150 Brabender units) and low dough development time).

A reomixer, a mixer similar to the typical mixograph, is used in cereal research to measure a range of rheological parameters that relate to the behavior of dough during bread making. In general, strong doughs have long mixing times (RM6), high peak heights (RM8), and wide bandwidths at 10 min (RM11) [41]. The reomixer properties of two generations of triticales from consecutive years of cultivation are presented in Table 4. (These generations represent vegetative seasons with different climatic conditions; the first year was typically dry, whereas the second was wet.) The reomixer analysis showed that the introgressive triticale lines had significantly (*p* < 0.05) higher values for five major reomixer parameters (IHTP, RM4, RM6, RM8, and RM11) than the triticale lines. For example, the mean values of the peak time (RM6) and area under the line (IHTP) for the F7 and F8 generations of the *Triticale/T. monococcum* lines with the 1D/1A chromosome substitution increased by 1.7-fold and 3.0-fold, respectively, in comparison with the secondary triticale lines. These dough-mixing parameters are similar to those of flour from weak wheat cultivars (data not shown). The considerable increase in these parameters results from an increase in the amount of polymeric proteins in these lines. The reomixer curve values of the three different triticale groups are shown in order, Glu + Sec + D1 + Tm > Glu-Sec + D1 > Glu-Sec + Tm. The reomixer data support the positive effect of *T. monococcum* gluten proteins on dough mixing. The presence of the *Glu-D1* locus encoding the Dx5 + Dy10 subunits in the *Triticum/T. monococcum* lines additionally improved the mixing properties and dough functionality.

#### 2.4.2. Micro-Scale Extension Test

**Figure 5:**
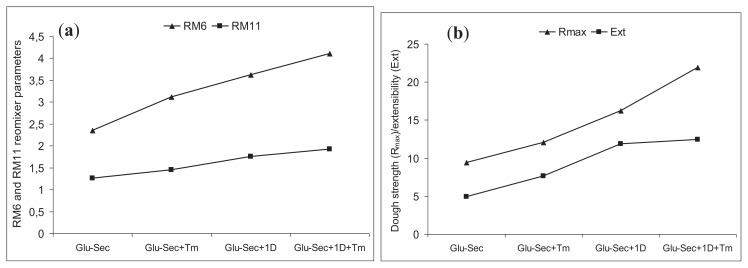
Comparison of means of selected reomixer (**a**) and extension test (**b**) parameters for the three group of introgressive triticale lines with additional HMW glutenin subunits [Glu-Sec + Tm (5.2 m + 14 m); Glu-Sec + 1D (5 + 10); Sec + Tm + 1D (5.2 m + 14 m and 5 + 10)] and secondary triticale (Glu-Sec) lines.

The parameters of the micro-scale extension test (large deformation dough rheology using the TA.XT2 texturometer with a Kieffer rig) include the resistance at peak extensibility (R_max_), maximum dough extension (Ext), and peak area under the curve (Area). In Table 4, the rheological properties of dough for the four triticale groups from two generations are presented. Significant differences in the R_max_, Ext, and Area were found in samples with various HMW glutenin subunit compositions (*p* < 0.05). The mean values for R_max_ and Area ranged from 9.4 to 21.9 g, and 39.2 to 172.5, respectively. The secondary triticale lines (Glu-Sec group) showed very poor extension parameters, and the dough prepared from these lines was very sticky. The absence of the wheat D genome, which was replaced by the rye R genome, in the secondary triticale considerably decreased the rheological properties of this crop. All the introgressive triticale lines (including those with the additional einkorn HMW-GS and/or wheat Dx5 + Dy10 subunits) (Figure 5b) had significantly higher values (*p* < 0.05) for all extension parameters than those of the secondary triticale lines. Particularly favorable rheological parameters were exhibited by the *Triticale/T. monococcum* lines expressing Dx5 + Dy10 subunits. The mean R_max_ and Area of these lines increased by 2.3-fold and 4.4-fold, respectively, in comparison with the secondary triticale lines. These mixing data confirm the earlier conclusion of Garg and coworkers [42], *i.e.*, that chromosome 1A has a small or negative overall effect on dough strength when the Glu-*1Ac* (AxNull) allele is present.

An increase in Ext was observed both in the lines containing the *T. monococcum* genome (Glu-Sec + Tm) and in the lines with the 1D/1A chromosome substitution. However, a significant increase in these parameters was not detected in the introgressive lines expressing both types of HMW glutenin subunit (Figure 5b).

A significantly lower R_max_/Ext ratio (*p* < 0.05) was observed in the lines with the 1D/1A substitution (Glu-Sec + 1D) compared to the other lines. The decrease in this ratio results from an increase in the HMW/LMW-GS ratio, which has a strong effect on the aggregation and polymerization properties of glutenin macropolymer (GMP) granules at all stages of dough mixing [43–45]. Don and coworkers [43] hypothesized that glutenin particles in GMPs can change shape due to mixing factors, so the distribution of glutenin particles plays an important role in dough rheology. In the presence of the einkorn A^m^ genome, a significant reduction of the R_max_/Ext ratio was not observed in the introgressive triticale lines. On the basis of these results, we speculate that the structure of both the x and y subunits in *T. monococcum* (for example, the number and position of cysteine residues in the gluten polymer and the sizes of the repetitive domains) may be responsible for the increase in dough strength and other rheological parameters. However, in future studies, the influence of the presence of the *Gli-D1*, *Glu-D3*, *Gli-A^m^*, and *Glu-A^m^3* loci on the rheological properties and the final quality of introgressive *Triticale/T. monococcum* lines should also be considered.

## 3. Experimental Section

### 3.1. Plant Materials

**Figure 6:**
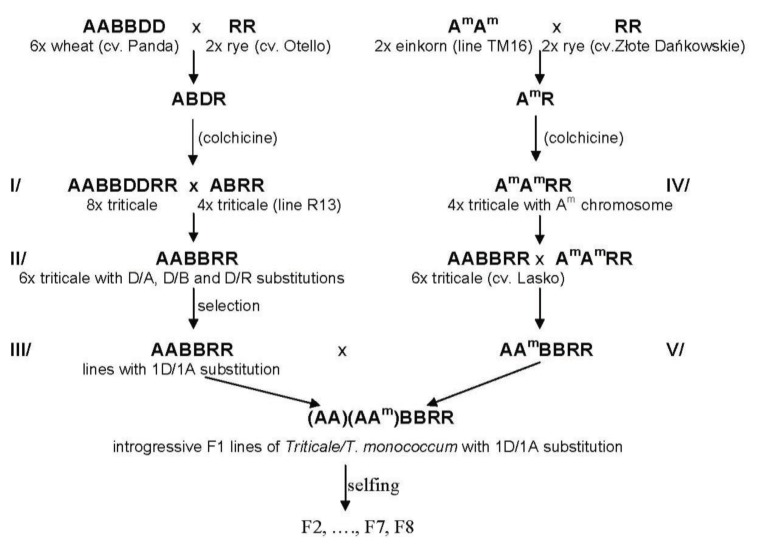
Crossing scheme to introduce wheat 1D and einkorn A^m^ chromosomes into hexaploid triticale.

The plant materials consisted of 36 secondary F7 and F8 hexaploid winter triticale lines containing the A^m^ genome from *Triticum monococcum* ssp. *monococcum* var. *macedonicum* Papag and a 1D/1A chromosome substitution. These genotypes were developed by crossing an initial octoploid triticale, designated “I/” (Figure 6), and derived from the hexaploid wheat (*Triticum aestivum* L.) cultivar Panda, which produced HMW glutenin subunit 5 + 10, and rye (*Secale cereale*) cv. Otello [46]. Hexaploid triticale with various D/A, D/B, and D/R substitutions, designated “II/” (Figure 6), were obtained by polyploid degree reduction during the crossing of octoploid triticale with various tetraploid triticale lines [47,48]. Based on cytological analyses, 42–44 chromosome lines with a single 1D/1A substitution were selected among the progeny and designated “III/”. The secondary hexaploid lines were developed by crossing the above hexaploid triticale lines with the hexaploid triticale containing the introgression of *T. monococcum* chromatin and were designated “IV/” and “V/” [15,48].

The 36 selected F7 and F8 generations of the secondary hexaploid triticale (AA^m^BBRR) with a 1D/1A chromosome substitution, two standard triticale varieties, and one wheat variety (cv. Lasko and cv. Gabo and cv. Panda, respectively), were grown in light brown soil for two consecutive seasons in the experimental field of the Institute of Plant Genetics, Polish Academy of Science, located in Cerekwica, approximately 30 km to the north of Poznań. The experiment was established using a complete block design with two replicates in 5 m^2^ plots, with a row spacing of 12.5 cm. The wheat was sown at the end of September and harvested during the first ten days of August. Fertilizer was applied at rates of 80 kg/ha N, 120 kg/ha K and 70 kg/ha P prior to sowing and 65 kg/ha N during springtime.

### 3.2. Identification of D-Genome Chromosomes Using FISH and GISH

Fluorescence/genomic *in situ* hybridization (FISH/GISH) was successfully used to detect the D-genome chromosomes in given hybrids. The root-tip chromosome preparations were made according to Pijnacker and Ferwerda [49]. The probes and the competitor DNA were generated from total genomic DNA of *Aegilops tauschii*, *Ae. speltoides*, *T. monococcum*, and triticale cv. Secundo. Genomic *in situ* hybridization (GISH) was used as a screening method to detect the alien (D genome) chromatin and chromosome rearrangements among different genomes. Total *Aegilops tauschii* genomic DNA was used as a probe; it was labeled by nicktranslation with digoxigenin-11-dUTP for the screening, or digoxigenin-11-dUTP and tetramethyl-rhodamine-5-dUTP (Roche) (1:1 ratio) for the identification process. Fluorescein isothiocyanate conjugated (FITC) anti-digoxigenin antibodies (Roche) were used for the immunodetection of the digoxigenated probes. The total *Triticum monococcum* genomic DNA probe (digoxigenin-11-dUTP) and the total *Secale cereale* genomic DNA probe (tetramethyl-rhodamine-5-dUTP) were used to categorize the “A” and “R” subgenomes, respectively. Unlabeled DNA from triticale cv. Secundo (AABBRR) and *Aegilops speltoides* (BB) were sheared by autoclaving and used as blocking DNA for the screening/identifying GISH. Fluorescence *in situ* hybridization (FISH) was carried out to identify chromosome sets using the ribosomal DNA probes 5S rDNA (pTa794, labeled with tetramethyl-rhodamine-5-dUTP) and 35S rDNA (digoxigenin-11-dUTP), and the repetitive DNA probes pSc119.2 (digoxigenin-11-dUTP) and pAs1 (tetramethyl-rhodamine-5-dUTP). The preparations were mounted and counterstained in Vectashield containing 4′,6-diamidino-2-phenylindole (DAPI) or propidium iodide. The GISH/FISH images were acquired using an Olympus XM10 CCD camera with an Olympus BX 61 epifluorescence microscope and processed using Cell-F imaging software (version 3.1; Olympus Soft Imaging Solutions GmbH: Münster, Germany) and Micrografx Picture Publisher software (version 8.1; Micrografx Inc.: Richardson, TX, USA).

### 3.3. Agronomic Traits and Flour Quality Determination

The single kernel weight was estimated using a 250-kernel subsample from each plot.

Grain samples were tempered to 14% moisture, and 150 grams of seed from each plot were milled to flour with a Brabender Quadrumat Junior experimental mill (Brabender, Duisburg, Germany).

Flour protein, moisture, and gluten content, and Zeleny sedimentation volume (ZSL) in the triticale flour were determined by near-infrared reflectance spectroscopy using a Diode Array 7200 NIR spectrophotometer (Perten Instruments AB, Huddinge, Sweden) and expressed on a 14% moisture basis. The measurements were calibrated according to cultivated triticale samples.

The Hagberg falling number (HFN) was measured using a Perten Instruments Falling Number 1800 machine calibrated to ISO-standard 3039 and ICC Standard No. 107 [50] along with 7 g of flour with an adjusted moisture content of 15%.

### 3.4. Extraction of HMW Glutenin and Secalin Subunits

Extraction of HMW-SS and HMW-GS was performed based on the method of Salmanowicz [34]. In total, 150 mg of flour was used for sequential extraction of albumins and globulins (two 5-min extractions with 0.4 M NaCl + 0.067 M HKNaPO_4_ (pH 7.6) at RT), gliadins (two 10-min extractions using 70% (*v*/*v*) ethanol), monomeric prolamins (two 15-min extractions using 50% isopropanol), and glutenins (a single extraction for 30 min with 0.05 M Tris-HCl buffer (pH 7.5) containing 50% *v*/*v* 1-propanol, 2 M urea and 1% *w*/*v* DTT at 60 °C under nitrogen). Each extraction was performed with vortexing, followed by centrifugation at 15,000× *g* for 8 min, except for glutenin, which was centrifuged for 10 min. The glutenin extract was used as the initial material for HMW glutenin and secalin separation. HMW proteins that were alkylated with 4-vinylpyridine were precipitated from the glutenin extract by addition of 1-propanol to a final concentration of 62% *v*/*v*. Precipitated HMW subunits were redissolved in a solution containing 40% *v*/*v* acetonitrile and 0.1% *w*/*v* trifluoroacetic acid. All samples were used for analyses within 24 h after extraction. The extracts were filtered through a 0.45 mm PVDF membrane before the CZE analysis. Three protein separations were performed for each analytical assay.

### 3.5. Assessment of HMW Glutenin and Secalin Subunits/Genotypes

Generally, HMW glutenin and secalin subunits were assayed using SDS-PAGE and capillary zone electrophoresis (CZE). Additionally, several primer sets for polymerase chain reaction (PCR), previously reported to be diagnostic for HMW glutenin genes, were used.

#### 3.5.1. Separation by SDS-PAGE

Alkylated HMW glutenin subunits were separated by electrophoresis in vertical SDS-PAGE gel on a Protean II xi cell unit (Bio-Rad, Hercules, CA, USA) using the discontinuous Tris-HCl-glycine buffer system of Laemmli [51]. Ten microliters of protein sample were loaded onto the upper 4.5% gel and electrophoresis was performed on 11.5% (*w*/*v*, C, 1.05%) polyacrylamide in resolving solution at 240 V for 45 min after the tracking dye migrated off the gel. The gels were stained overnight with Coomassie Brilliant Blue G-250. The designation of the wheat HMW-GS and rye HMW-SS in secondary hexaploid triticale was performed according to McIntosh *et al*. [52].

#### 3.5.2. Separation of HMW Proteins by CZE

The HMW protein separations were carried out according to Salmanowicz [34], with some modifications. A solution containing 75 mM IDA, 0.15% PEO with a molecular weight of 8,000,000, 26 mM SB3-12, and 20% (*v*/*v*) acetonitrile (AcN) was used as the separation buffer. A buffer containing 0.1 M IDA, 0.2% (*w*/*v*) PVP (M_r_ 360,000), 0.05% (*w*/*v*) HPMC, and 20% (*v*/*v*) AcN was used as the polymer solution for dynamic coating of the capillary wall. All solutions and buffers were filtered through a 0.2 mm syringe filter. The separations were performed with a constant voltage of 10 kV at 40 °C on a Beckman-Coulter P/ACE System MDQ capillary electrophoresis instrument (Beckman Coulter, Fullerton, CA, USA). The system was equipped with a built-in 0–30 kV high-voltage power supply, diode array detector, and 32 Karate ver. 8.0 software (Beckman Coulter, Fullerton, CA, USA). The separations were carried out using uncoated fused silica capillaries (Polymicro Technologies, Phoenix, AZ, USA) with an internal diameter of 50 mm and 31.2 cm in total length, and a detection window was created at 21 cm from the capillary inlet. The capillary was equilibrated with running buffer for 5 min (0.3 MPa) before each sample injection. To ensure good reproducibility, after each separation, the capillary was rinsed stepwise with 0.1 N HCl (0.2 MPa for 4 min), Milli-Q water (0.2 MPa, 1 min), and then the coating polymer solution (0.25 MPa, 3 min). Samples were injected hydrodynamically under low pressure (0.5 psi (3.447 × 10^−3^ MPa)) for 8 s into the anodic end. Proteins were detected by UV absorbance at 200 nm. Three protein separations were performed by CZE for each analytical assay. The particular HMW-SS and HMW-GS were identified by CZE through comparison of single and mixed samples using the wheat cultivar Panda and two standard triticale cultivars, Lasko and Gabo.

### 3.6. Molecular Markers—DNA Extraction and PCR Amplification

Genomic DNA was extracted from the leaves of single plants, according to Salmanowicz and Dylewicz [31]. Two pairs of primers were used for the amplification of DNA fragments corresponding to the x-type genes present at the *Glu-A1* and *Glu-D1* loci. The oligonucleotides used as primers were synthesized according to published data (Table 1), and were purchased from Sigma-Genosys (Bremen, Germany).

PCR analyses were performed in a PTC-200 thermal cycler (Bio-Rad, Hercules, CA, USA) with a heated lid in a final volume of 25 L. The single PCR reaction mixture contained 1× buffer (Qiagen GmbH, Hilden, Germany), 2 mM MgCl_2_, 300 M of each dNTP, 0.2 M of each primer, 50 ng genomic DNA, and 0.5 unit HotStar DNA Polymerase (Qiagen GmbH, Hilden, Germany). The next step was checked at annealing temperature in the range 58–60 °C for 40 s to 1 min, extension time 10 min at 72 °C, and number of cycles, 35. The PCR products were also separated in ethidium bromide-stained 1.0% (*w*/*v*) agarose gels run in 1× TBE buffer and exposed to UV light to visualize DNA fragments.

### 3.7. Rheological Study

#### 3.7.1. Quality of Dough Mixing Properties

Farinograms were obtained with a type E micro-farinograph (Brabender, Duisburg, Germany) with a Mixer S10 (10 g flour) and a test profile corresponding to a procedure described in the AACC Standard 54–21.02 [53] and ICC 115/1 [50] for the determination of the water absorption of flour. Each of the farinograph was run for 20 min. Two to three replicates were used to achieve farinograms that had a maximum resistance centered on the 500-BU line.

Dough mixing properties were determined with a computerized Bohlen Reomixer (Reologen i Lund AB, Lund, Sweden), which is a planetary pin mixer similar to the Mixograph (National Manufacturing, Lincoln, NE, USA) with a 10 g flour capacity, a mixing speed of 88 rpm and data recording at 10 points/s [41]. Mixing was performed for 10 min at 30 °C. All measurements were carried out using a mix time of 10 min. Samples were supplemented with 5.84–6.25 mL of a 2% NaCl solution, depending on the protein content in the sample. At the end of mixing, 16 pre-selected parameters were automatically extracted from the Reomixer trace. Five of these parameters, described in more detail by Salmanowicz *et al*. [54], were selected, covering all phases of dough development and describing all basic rheological aspects of mixing characteristics, area under the center line up to the peak (IHTP), time 1–2 (RM4), peak time (RM6), peak height (RM8), and bandwidth at 10 min (RM11).

#### 3.7.2. Micro-Scale Extension Test

A TA.XT2 texture analyzer (Stable Micro Systems Ltd., Surrey, UK), equipped with a Kieffer dough and gluten extensibility rig, was used for the dough extensibility measurements. Dough was prepared by mixing 10 g of flour and 0.2 g of sodium chloride (NaCl) with the appropriate amount of water in a 10 g reomixer for 6 min at 30 °C. Dough collected from the reomixer was rolled gently into a ball, placed in a plastic container and kept in the proofing chamber (at 30 ± 1 °C) for 20 min. After the resting period, dough was shaped into an ellipsoid form and was pressed into Teflon forms to give strands/strips of 53 × 4 × 4 mm. After 40 min of resting in a desiccator, in a water-saturated atmosphere, the strands were measured at a hook speed of 3.3 mm/s and a trigger force of 1 g using a 5-kg load cell. The parameters obtained from the Kieffer force-distance curves were the maximum peak force (resistance at peak extensibility, (Rmax)), maximum dough extension (extensibility, (Ext)), and the area under the force by distance curve (Area). Five replicates were carried out for each test and the corresponding averages were calculated.

### 3.8. Statistical Analysis

Data were analyzed using the STATISTICA statistical software package (version 10.1 PL; StatSoft Polska, Kraków, Poland). Initially, exploratory association analysis was performed on the rheological properties (farinograph and Kieffer rig parameters) using combining data from the 2-year period (generations F7 and F8). This analysis revealed significant (*p* < 0.001) genotype-environment interactions between HMW-GS and -SS composition and vegetation seasons (data not shown). Thus, the data presented in this study were analyzed separately by year. The analysis of variance (ANOVA) and the least significant difference pairwise comparisons of means were used to determine significant differences. Statistical significance was declared at *p* < 0.05.

## 4. Conclusions

The results obtained in this study indicate that cultivated einkorn gluten proteins have a positive effect on the end-product quality of hexaploid winter triticale. Additionally, replacement of chromosome 1A, which contains the Glu-*1Ac* (AxNull) allele, Glu-*1Ac* (AxNull), with chromosome 1D improves a number of physicochemical grain traits and rheological dough properties. Introgressive *Triticale/T. monococcum* lines exhibit increased protein and gluten content, Zeleny sedimentation value, and water absorption capacity. These lines are characterized by favorable dough-mixing properties and have a significantly higher maximum resistance to extension (R_max_) and dough extensibility than typical triticale lines. Generally, the rheological parameters of flour from *Triticale/T. monococcum* with a 1D/1A chromosome substitution are similar to those of flour from weak wheat cultivars. However, there remains a problem with the bread-making quality of triticale due to low Hagberg falling numbers (below 100 s), dough development time, and high values of degree of dough softening (above 150 Brabender units). In summary, from the data presented here, we conclude that the cultivated einkorn HMW glutenin subunit composition, in concert with triticale HMW-GS and the wheat Dx5 + Dy10 subunits introduced by a 1D/1A substitution, has considerable potential to improve gluten polymer interactions and is a valuable genetic resource for triticale quality improvement.

## Figures and Tables

**Table 1:** Sets of allele-specific markers used for identification of high molecular weight (HMW) glutenin genes in triticale genotypes.

Primer set	HMW-GS encoded by DNA fragment	Expect size of DNA fragment	Forward and reverse primer sequences 5′→3′	References
GS	I Dx5/Dx2	450 bp	F: GCCTAGCAACCTTCACAATC R: GAAACCTGCTGCGGACAAG	[28]
GS II	AxNull	920 bp	F: ACGTTCCCCTACAGGTACTA R: TATCACTGGCTAGCCGACAA	[29]
GS III	By20^*^	750 bp	F: TTCTCTGCATCAGTCAGGA R: AGAGAAGCTGTGTAATGCC	[30,31]

**Table 2:** Characterization of the analyzed material on the basis of HMW glutenin and secalin subunit composition.

Designation of triticale groups	Loci					No. of lines

	*Glu-A1*	*Glu-A1^m^*	*Glu-B1*	*Glu-D1*	*Sec-3*	
Glu-Sec	Null	-	6.8 + 20^*^	-	2r + 5.3r	14
Glu-Sec+1D	Null	-	6.8 + 20^*^	5 + 10	2r + 5.3r	3
Glu-Sec+Tm	Null	5.2 m + 14 m	6.8 + 20^*^	-	2r + 5.3r	16
Glu-Sec+Tm+1D	Null	5.2 m + 14m	6.8 + 20^*^	5 + 10	2r + 5.3r	3

Total no. of lines						36

**Table 3:** Physicochemical properties of four winter triticale groups with various HMW glutenin subunit compositions at two generations (F7 and F8) from field experiments, harvested in 2010 and 2011, respectively.

Quality characteristics	Glu-Sec	Glu-Sec + Tm	Glu-Sec + 1D	Glu-Sec + Tm + 1D
**F7** (2009/2010)				
				
1000-kernel weight, g	41.8 ^a^,*	38.9 ^b^	39.6 ^b^	36.1 ^c^
Protein content, 14% mb	10.6 ^a^	11.5 ^b^	11.8 ^b^	12.0 ^b^
Gluten content, %	15.5 ^a^	18.4 ^b^	19.2 ^b^	19.8 ^b^
Moisture contain, %	11.1 ^a^	11.3 ^a^	10.9 ^a^	11.0 ^a^
Ash content, %	0.75 ^a^	0.84 ^a^	0.79 ^a^	0.86 ^a^
ZSV, mL	22.7 ^a^	26.1 ^b^	27.5 ^b^	27.9 ^b^
Hagberg falling number, s	66 ^a^	70 ^a^	69 ^a^	71 ^a^

**F8** (2010/2011)				
				
1000-kernel weight, g	41.2 ^a^	38.8 ^b^	40.1 ^c^	37.8 ^b^
Protein content, 14% mb	10.3 ^a^	11.4 ^b^	11.6 ^b^	11.6 ^b^
Gluten content, %	15.1 ^a^	17.1 ^c^	18.0 ^c^	18.4 ^c^
Moisture contain, %	12.6 ^a^	12.1 ^a^	12.2 ^a^	12.6 ^a^
Ash content, %	0.81 ^a^	0.88 ^a^	0.78 ^a^	0.91 ^a^
ZSV, mL	21.1 ^a^	24.5 ^b^	25.9 ^b^	26.7 ^b^
Hagberg falling number, s	64 ^a^	68 ^a^	67 ^a^	70 ^a^

* Different letters means significant difference at the *p* < 0.05 level within the same parameter for particular generations;

ZSV, Zeleny sedimentation value.

**Table 4:** Dough rheological properties of the four winter triticale groups from the F7 and F8 generations from field experiments, harvested in 2010 and 2011, respectively.

Character	Glu-Sec	Glu-Sec + Tm	Glu-Sec + 1D	Glu-Sec + 1D + Tm
**F7** (2009/2010)				
				
**Micro-Farinograph parameters**				
Water absorption (g/100 g)	66.6 ^a^,*	68.1 ^b^	67.8 ^b^	68.9 ^b^
Dough development time (min)	1.0 ^a^	1.4 ^a^,^b^	1.4 ^b^	1.8 ^b^
Dough stability (min)	1.2 ^a^	1.7 ^b^	1.6 ^b^	2.2 ^c^
Degree of softening (BU)	196 ^a^	164 ^b^	172 ^b^	161 ^b^
**Reomixer parameters**				
Area under line (IHTP)	6.7 ^a^	11.6 ^b^	13.6 ^b^	22.5 ^c^
Time 1–2 (RM4) (min)	1.21 ^a^	1.54 ^a^,^b^	1.98 ^b^	3.10 ^c^
Peak time (RM6) (min)	2.59 ^a^	3.50 ^b^	3,84 ^b^	4.41 ^c^
Peak height (RM8) (cm)	1.52 ^a^	2.24 ^b^	3.13 ^c^	4.30 ^d^
Bandwidth at 10 min (RM11)	1.28 ^a^	1.46 ^b^	1.79 ^b^	1.97 ^b^
**SMS/Kieffer rig parameters**				
Resistance at peak extensibility (R_max_)	10.0 ^a^	12.5 ^b^	16.2 ^c^	23.4 ^d^
Max.dough extension (Ext) (mm)	5.1 ^a^	8.1 ^b^	12.2 ^c^	12.9 ^c^
Area under R_max_*vs*. Ext curve (Area)	41.9 ^a^	79.5 ^b^	129.1 ^c^	188.6 ^d^
R_max_/Ext (g/mm)	1.90 ^a^	1.58 ^b^	1.32 ^b^	1.81 ^a^

**F8** (2010/2011)				
				
**Micro-Farinograph parameters**				
Water absorption (g/100g)	65.1 ^a^	67.9 ^b^	67.2 ^b^	68.1 ^b^
Dough development time (min)	0.9 ^a^	1.3 ^a^	1.4 ^a^	1.7 ^b^
Dough stability (min)	1.1 ^a^	1.6 ^b^	1.7 ^b^	1.9 ^b^
Degree of softening (BU)	211 ^a^	178 ^b^	181 ^b^	168 ^b^
**Reomixer parameters**				
Area under line (IHTP)	6.5 ^a^	10.0 ^b^	12.1 ^b^	21.3 ^c^
Time 1–2 (RM4) (min)	1.06 ^a^	1.46 ^b^	1.79 ^b^	2.83 ^c^
Peak time (RM6) (min)	2.12 ^a^	2.74 ^b^	3.41 ^c^	3.80 ^c^
Peak height (RM8) (cm)	1.78 ^a^	2.41 ^b^	2.76 ^b^	4.12 ^c^
Bandwidth at 10 min (RM11)	1.23 ^a^	1.44 ^a^	1.71 ^b^	1.88 ^b^
**SMS/Kieffer rig parameters**				
Resistance at peak extensibility (R_max_)	8.8 ^a^	11.6 ^b^	16.3 ^c^	20.4 ^d^
Max. dough extension (Ext) (mm)	4.7 ^a^	7.2 ^b^	11.6 ^c^	12.0 ^c^
Area under R_max_*vs.* Ext curve (Area)	36.5 ^a^	67.9 ^b^	114.3 ^c^	156.4 ^d^
R_max_/Ext (g/mm)	1.87 ^a^	1.61 ^a^	1.36 ^b^	1.70 ^a^

* Different letters means significant difference at the *p* < 0.05 level within the same parameter for particular generations;

BU, Brabender units.
